# Cardiovascular subphenotypes in patients with COVID‐19 pneumonitis whose lungs are mechanically ventilated: a single‐centre retrospective observational study

**DOI:** 10.1111/anae.15700

**Published:** 2022-03-03

**Authors:** M. Chotalia, M. Ali, J. E. Alderman, J. M. Patel, D. Parekh, M. N. Bangash

**Affiliations:** ^1^ Department of Anaesthesia and Critical Care Medicine Queen Elizabeth Hospital Birmingham UK; ^2^ Department of Anaesthesia and Critical Care Medicine Queen Elizabeth Hospital UK; ^3^ Department of Anaesthesia and Critical Care Medicine Queen Elizabeth Hospital UK

**Keywords:** acute respiratory distress syndrome, right ventricular dysfunction, right ventricular failure, transthoracic echocardiography

## Abstract

Unsupervised clustering methods of transthoracic echocardiography variables have not been used to characterise circulatory failure mechanisms in patients with COVID‐19 pneumonitis. We conducted a retrospective, single‐centre cohort study in ICU patients with COVID‐19 pneumonitis whose lungs were mechanically ventilated and who underwent transthoracic echocardiography between March 2020 and May 2021. We performed latent class analysis of echocardiographic and haemodynamic variables. We characterised the identified subphenotypes by comparing their clinical parameters, treatment responses and 90‐day mortality rates. We included 305 patients with a median (IQR [range]) age 59 (49–66 [16–83]) y. Of these, 219 (72%) were male, 199 (65%) had moderate acute respiratory distress syndrome and 113 (37%) did not survive more than 90 days. Latent class analysis identified three cardiovascular subphenotypes: class 1 (52%; normal right ventricular function); class 2 (31%; right ventricular dilation with mostly preserved systolic function); and class 3 (17%; right ventricular dilation with systolic impairment). The three subphenotypes differed in their clinical characteristics and response to prone ventilation and outcomes, with 90‐day mortality rates of 22%, 42% and 73%, respectively (p < 0.001). We conclude that the identified subphenotypes aligned with right ventricular pathophysiology rather than the accepted definitions of right ventricular dysfunction, and these identified classifications were associated with clinical outcomes.

## Introduction

In patients with COVID‐19 pneumonitis, right ventricular dysfunction is common and associated with mortality [1, 2, 3, 4], which makes its prevention and management a potentially important therapeutic target [5, 6]. However, right ventricular dysfunction is inconsistently defined by most studies using either right ventricular dilation (with septal dyskinesia [7, 8] or venous congestion [9]) or right ventricular systolic impairment [10, 11]. Fulfilment of either criterion is inconsistently associated with mortality [12]. This is unsurprising, given that right ventricular function is assessed in a binary fashion using only a few variables, whose measurements are compounded by the complex geometry of the right ventricle and by cut‐off values that are not validated in COVID‐19 pneumonitis. Furthermore, although the right ventricle is assessed in isolation, it is connected in series to the left ventricle with the effect of right ventricular failure on left ventricular function seldom considered. A definition that captures the pathophysiology of the disease, incorporates global cardiovascular function and aligns with patient outcomes is required.

Haemodynamic interventions are applied broadly to patients with acute respiratory distress syndrome (ARDS) [13]. However, the mechanisms of cardiovascular dysfunction are varied [14] and this underlying heterogeneity may impede the delivery of targeted therapies. Latent class analysis can be used to identify discrete yet unobserved classes by clustering patients with similar patterns of observed variables together, generating homogenous sub‐groups from heterogeneous populations [15]. In non‐COVID‐19 ARDS, the approach has been used to leverage many blood and physiological variables to consistently identify two subphenotypes (hyper‐ and hypo‐inflammatory ARDS) with distinct clinical, biological, treatment and outcome characteristics across multiple studies [16, 17]. Recently, similar techniques using haemodynamic parameters have described five cardiovascular clusters in septic shock [14]. A comparable approach has not been performed in COVID‐19 pneumonitis and could present an unbiased, non‐binary and multimodal way of characterising circulatory failure in patients with this disease. This could overcome the limitations of current cardiology‐based definitions.

The aim of this study was to perform latent class analysis of transthoracic echocardiographic and haemodynamic variables to identify and characterise cardiovascular subphenotypes in COVID‐19 pneumonitis. A secondary aim was to compare the outcomes and response with right ventricular protective measures of identified subphenotypes.

## Methods

This retrospective single‐centre observational study was approved by a local NHS Research Ethics Committee. We included patients with COVID‐19 pneumonitis whose lungs were invasively ventilated and who underwent transthoracic echocardiography at the Queen Elizabeth Hospital, Birmingham, UK, between March 2020 and May 2021. Patient management was protocolised as outlined previously [4]. Patients who: received extracorporeal membrane oxygenation; did not meet Berlin ARDS criteria; were not receiving invasive positive pressure ventilation at the time of echocardiography; or who had pre‐existing left or right ventricular dilation or systolic dysfunction, were not studied.

We recorded clinical and laboratory data from the time of the echocardiographic examination. If prone positioning was performed on the day of the examination, we recorded clinical variables before and 6 h after changing to the prone position. We calculated: chest radiograph opacification [18]; ARDS severity [19]; dead space fraction [20]; dynamic compliance [4]; and vasopressor dose [21], according to established definitions.

A transthoracic echocardiogram was requested at the discretion of the treating clinician after documentation of an elevated high‐sensitivity troponin‐I (> 14 ng.l^‐1^). The imaging protocol used has been outlined previously [4]. Briefly, a modified British Society of Echocardiography level‐1 protocol was performed by echocardiographers or clinicians with level‐2 accreditation [22]. Right ventricular end‐diastolic area, right ventricular end‐systolic area and left ventricular end‐diastolic area were recorded in triplicate offline by two independent observers blinded to the clinical data and accredited in critical care echocardiography. A right:left ventricular end‐diastolic area of >0.6 defined right ventricular dilation, and right ventricular fractional area change < 35% or tricuspid annular plane systolic excursion < 17 mm defined right ventricular systolic impairment. Left ventricular ejection fraction was assessed visually (British Society of Echocardiography level‐1 guidance). We estimated the probability of pulmonary hypertension as per European guidelines [23]. We also recorded inferior vena cava diameter and collapsibility. We collected data on the first echocardiographic examination for included patients.

We tested all continuous echocardiographic and haemodynamic variables for normality and those with a skewed distribution underwent log or square root transformation. We placed continuous variables on a z‐scale and examined correlation using the Pearson correlation coefficient. We excluded any one of two collinear variables (coefficient > 0.5) and performed sensitivity analyses with inclusion of each of the excluded variables. We included vasopressor dose as an ordinal variable (0, > 0 – < 0.1, ≥ 0.1 mcg.kg^‐1^.min^‐1^), due to a persistent non‐normal distribution, despite transformation. We handled missing data using the full information maximum likelihood function.

We evaluated the best fit of models ranging from one to five classes using Akaike information criteria, Bayesian information criteria, Vuong‐Lo–Mendell–Rubin likelihood ratio test, entropy and class size. We assessed local dependence within classes through bivariate residuals, with sensitivity analyses performed on excluded co‐dependent variables. We allocated classes based on a posterior probability of class assignment >50%. Entropy values, which are a measure of class separation, were considered acceptable if above 0.8 [24]. We performed all latent class analyses using Latent Gold (v 6.0, Statistical Innovations, Arlington, MA, USA). Continuous variables were analysed using a Kruskal–Wallis test. Ordinal variables were compared using a Chi‐square test. We considered a p value < 0.05 to be statistically significant and all tests were two‐sided. This was a pragmatic study and we did not perform post‐hoc power calculations to determine a sample size. We assessed intra‐ and inter‐observer variation of echocardiographic measurements using the coefficient of variation. We performed all other analyses other than latent class analysis using GraphPad Prism (v9.1, GraphPad, San Diego, CA, USA).

## Results

**Figure 1:**
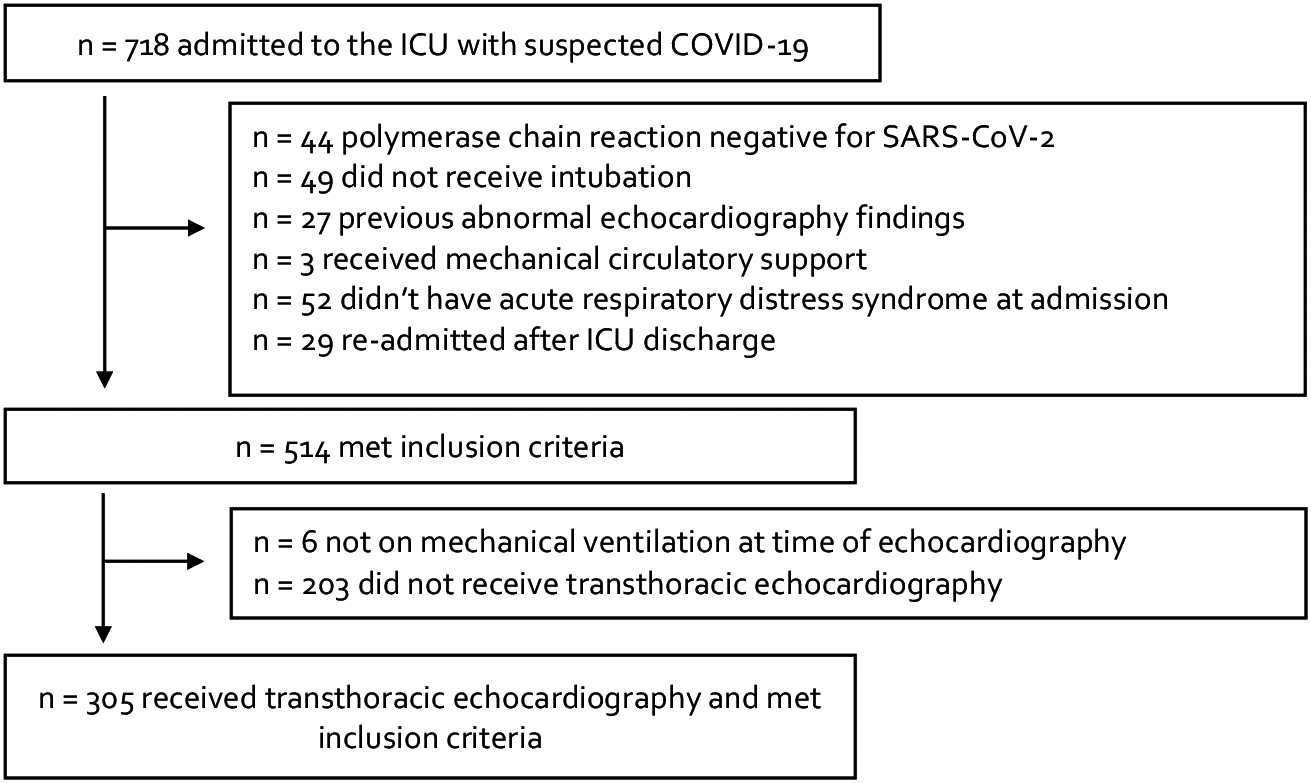
Flow chart for inclusion of patients in the study.

We included 508 patients with COVID‐19 pneumonitis, of whom 305 (60%) underwent echocardiography (Fig. 1, Table 1 and online Supporting Information Table S1). Of these, 248 (81%) echocardiographic examinations occurred within 48 h of cardiovascular dysfunction. For right ventricular fractional area change and right:left ventricular end‐diastolic area, intra‐observer variability was 3.2% and 3.9%, and inter‐observer variability was 6.8% and 5.5%, respectively.

**Table 1:** Comparison of baseline characteristics and clinical variables in cardiovascular subphenotypes. Values are mean (SD), number (proportion) or median (IQR [range])

	All n = 305	Class 1 n = 158	Class 2 n = 95	Class 3 n = 52	p value
Age; y	57 (12)	55 (13)	60 (12)	58 (10)	0.010
Sex; male	219 (72%)	104 (66%)	74 (78%)	40 (77%)	0.076
Day of transthoracic echocardiogram	8 (5–13 [1–31])	8 (4–13 [1–26])	9 (5–14 [1–31])	7 (4–12 [1–24])	0.482
Acute respiratory distress syndrome severity					0.014
Mild	54 (18%)	36 (23%)	12 (13%)	6 (12%)	
Moderate	199 (65%)	105 (67%)	61 (64%)	33 (63%)	
Severe	52 (17%)	17 (11%)	22 (23%)	13 (25%)	
PaO_2_:F_I_O_2_	20 (16–25 [7–39])	22 (17–27 [10–39])	19 (14–24 [8–39])	18 (13–22 [7–39])	< 0.001
PaCO_2_; kPa	7 (6–9 [4–15])	7 (6–9 [4–13])	8 (7–10 [4–13])	7 (7–10 [6–15])	0.031
pH	7.34 (7.28–7.39 [7.00–7.50])	7.34 (7.29–7.39 [7.10–7.50])	7.32 (7.27–7.37 [7.10–7.50])	7.31 (7.22–7.37 [7.00–7.40])	0.016
White blood cell count; x 10^9^ l^‐1^	13 (9–16 [0–44])	12 (9–16 [1–32])	13 (10–18 [1–39)	12 (8–16 [0–44])	0.188
C‐reactive protein; mg.ml^‐1^	132 (65–221 [5–554])	115 (59–189 [5–409])	148 (74–249 [5–449])	167 (76–266 [5–554])	0.001
Troponin; ng.l^‐1^	26 (9–161 [4–72,304])	7 (15–62 [4–1995])	26 (9–132 [4–2983])	132 (35–1109 [10–72,304])	< 0.001
D‐dimer; ng.ml^‐1^	1349 (617–3392 [104–42,230])	1035 (456–2680 [104–13,349])	1978 (855–4286 [113–30,093])	2498 (664–6403 [109–42,230])	0.002
Chest radiograph opacification score (0–16)	8 (6–10 [3–16])	8 (6–8 [3–14])	8 (6–10 [4–16])	9 (8–11 [4–16])	< 0.001
Dead space fraction	0.74 (0.65–0.81 [0.25–0.92])	0.72 (0.64–0.80 [0.25–0.92])	0.74 (0.65–0.80 [0.25–0.89)	0.78 (0.72–0.86 [0.32–0.92])	0.001
Dynamic compliance; ml.cmH_2_O^‐1^	28 (21–34 [10–77])	29 (21–38 [17–77])	26 (21–33 [15–61])	24 (19–32 [10–49])	0.019
Peak airway pressure; cmH_2_O	26 (21–30 [12–42])	25 (20–29 [12–30])	28 (22–30 [14–34])	29 (25–32 [20–42])	< 0.001
Positive‐end expiratory pressure; cmH_2_O	8 (6–10 [4–16])	8 (5–10 [4–14])	8 (6–10 [4–14])	8 (7–10 [4–16])	0.056
Urine output; ml.kg^‐1^.h^‐1^	0.60 (0.31–0.94 [0–2.70])	0.70 (0.46–1.0 [0–2.40])	0.58 (0.22–0.82 [0–2.70])	0.37 (0.14–0.66 [0–2.60])	< 0.001
Second vaso‐active agent	24 (8%)	3 (2%)	9 (10%)	12 (23%)	< 0.001
Prone ventilation	219 (72%)	108 (69%)	70 (74%)	40 (77%)	0.419
Neuromuscular blockade	269 (89%)	136 (86%)	85 (89%)	49 (92%)	0.261
Renal replacement therapy	154 (51%)	68 (43%)	53 (56%)	33 (64%)	0.018
90‐day mortality	113 (37%)	35 (22%)	40 (42%)	38 (73%)	< 0.001

Exclusion of variables due to collinearity and local dependence had little effect on model selection (see online Supporting Information Appendix S1). Eight variables were included in the final model: right:left ventricular end‐diastolic area; right ventricular fractional area change; left ventricular end‐diastolic area; tricuspid annular plane systolic excursion; left ventricular eccentricity index at end‐diastole; inferior vena cava diameter; left ventricular ejection fraction; and vasopressor dosage. The median (IQR [range]) proportion of missing data in these variables was 2 (0–4 [0–9]) %. Bayesian information criteria decreased from classes 1 to 3 but increased when classes 4 and 5 were added. Akaike information criteria decreased sequentially; however, the rate of decrease was less with the addition of classes 4 and 5 (Table 2). The three‐class model had an improved model fit (p < 0.001) and higher entropy compared with the two‐class model and was therefore judged to be the best fit. In the three‐class model, the median (IQR [range]) posterior probability of class assignment was 89 (78–98 [58–100]) %, 88 (77–96 [56–100]) % and 98 (89–100 [62–100]) % for classes 1–3, respectively, indicating strong class differentiation. While the Vuong‐Lo–Mendell–Rubin test demonstrated an improved model fit with the addition of a fourth class (p = 0.011), the increase in Bayesian information criteria and decreased reduction in Akaike information criteria with the addition of a fourth class resulted in rejection of this model.

**Table 2:** Fit‐statistics for one to five class models of latent class analysis.

Cluster	Likelihood ratio	Bayesian information criteria	Akaike information criteria	Maximum bivariate residual	Vuong‐Lo–Mendell–Rubin test	Entropy
1	−3213.9	6519.4	6459.9	56.7		1.00
2	−2988.7	6154.8	6039.5	8.9	0.000	0.81
3	−2936.2	6135.7	5964.5	5.8	0.000	0.81
4	−2896.9	6142.3	5915.4	6.0	0.011	0.72
5	−2868.2	6171.2	5888.5	7.7	0.042	0.75

In the three‐class model, 158 (52%) were assigned to class 1, 95 (31%) to class 2 and 52 (17%) to class 3 (Table 3). Class 3 was distinguished primarily by markedly increased right ventricle size and severely reduced systolic function compared with both classes 1 and 2. Vasopressor dose and inferior vena cava diameter were increased, as was the incidence of septal dyskinesia, small left ventricular size and hyperdynamic systolic function. When comparing classes 1 and 2, both had similar right ventricular systolic function, along with comparable left ventricular systolic function. However, class 2 had significantly increased right ventricular size, inferior vena cava diameter, left ventricular eccentricity index at end‐diastole and vasopressor requirements compared with class 1. These variables were still lower in class 2 than class 3. Class 1 had relative preservation of all cardiovascular parameters.

**Table 3:** Comparison of echocardiographic and haemodynamic variables in cardiovascular subphenotypes. Values are median (IQR [range]) or number (proportion).

	All n = 305	Class 1 n = 158	Class 2 n = 95	Class 3 n = 52	p value
**Right ventricle**					
End‐diastolic area index; cm^2.^m^‐2^	10 (8–12 [4–22])	9 (7–11 [4–14])	11 (9–13 [6–18])	13 (11–15 [6–22])	< 0.001
End‐systolic area index*;* cm^2^.m^‐2^	6 (5–8 [2–16])	5 (4–6 [2–11])	7 (6–8 [3–15])	10 (9–13 [4–16])	< 0.001
Right:left ventricular end‐diastolic area	0.61 (0.53–0.78 [0.30–1.40])	0.53 (0.45–0.59 [0.30–0.80])	0.72 (0.65–0.8 [0.50–1.00)	0.91 (0.85–1.0 [0.6–1.4])	< 0.001
Fractional area change; %	35 (27–44 [8–65])	40 (32–47 [25–65])	37 (30–43 [17–54])	21 (18–24 [7–34])	< 0.001
Tricuspid annular plane systolic excursion; mm	22 (19–25 [6–39])	23 (20–26 [14–39])	20 (18–23 [12–38])	16 (12–20 [6–25])	< 0.001
Dilation	159 (52%)	23 (15%)	84 (88%)	52 (100%)	< 0.001
Systolic impairment	149 (49%)	60 (38%)	37 (39%)	52 (100%)	< 0.001
Septal dyskinesia	85 (28%)	33 (21%)	19 (20%)	33 (64%)	< 0.001
Eccentricity index – diastole	1.03 (0.98–1.11 [0.70–1.70])	1.00 (0.97–1.03 [0.70–1.40])	1.09 (1.02–1.18 [0.70–1.40])	1.10 (0.98–1.22 [0.90–1.70])	< 0.001
Eccentricity index – systole	1.00 (0.98–1.10 [0.40–20])	1.00 (0.96–1.05 [0.40–1.40])	1.04 (1.00–1.11 [0.70–1.40])	1.10 (1.00–1.25 [0.90–2.00])	< 0.001
**Pulmonary hypertension probability**					<0.001
Low	54 (18%)	32 (20%)	17 (18%)	5 (10%)	
Intermediate	43 (14%)	16 (10%)	17 (18%)	10 (19%)	
High	42 (14%)	16 (10%)	9 (10%)	17 (33%)	
Unable to determine	166 (54%)	94 (60%)	52 (55%)	20 (39%)	
Tricuspid regurgitation velocity maximum; m.s^‐1^	2.8 (2.3–3.2 [1.1–4.3])	2.7 (2.3–3.1 [1.1–3.5])	2.6 (2.2–3.1 [1.1–3.9])	3.0 (2.8–3.4 [1.9–4.3])	0.010
**Left ventricle**					
Ejection fraction					0.002
Normal (55–70%)	156 (51%)	79 (50%)	61 (64%)	16 (31%)	
Depressed (< 55%)	32 (11%)	20 (13%)	5 (5%)	7 (14%)	
Hyperdynamic (> 70%)	117 (38%)	59 (37%)	29 (31%)	29 (56%)	
Ejection fraction; %	65 (60–75 [10–85])	65 (60–75 [15–80])	65 (60–75 [10–85])	75 (60–75 [10–85])	0.031
End‐diastolic area index; cm^2^.m^‐2^	16 (14–18 [8–28])	17 (15–19 [10–28])	15 (14–17 [10–24])	15 (13–17 [8–23])	< 0.001
Inferior vena cava diameter; cm	2 (2–2 [0–3])	2 (1–2 [0–3])	2 (2–2 [0–3])	2 (2–3 [1–3])	< 0.001
Collapsibility of inferior vena cava; %	29 (1–50 [0–100])	42 (15–54 [0–100])	32 (10–53 [0–100])	0 (0–8 [0–73])	< 0.001
**Haemodynamic**					
Mean arterial pressure; mmHg	74 (68–81 [49–110])	75 (70–85 [55–110])	73 (67–80 [49–109])	71 (65–75 [51–98])	< 0.001
Vasopressor use	192 (63%)	76 (48%)	75 (79%)	41 (79%)	< 0.001
Vasopressor dose; mcg.kg^‐1^.min^‐1^	0.05 (0.00–0.17 [0.00–1.00])	0.00 (0.00–0.08 [0.00–0.73])	0.10 (0.02–0.25 [0.00–1.00])	0.17 (0.06–0.33 [0.00–1.00])	< 0.001
Heart rate; beats.min^‐1^	88 (72–101 [42–135])	86 (72–97 [42–135])	89 (71–102 [49–129])	94 (78–102 [48–133])	0.147

**Figure 2:**
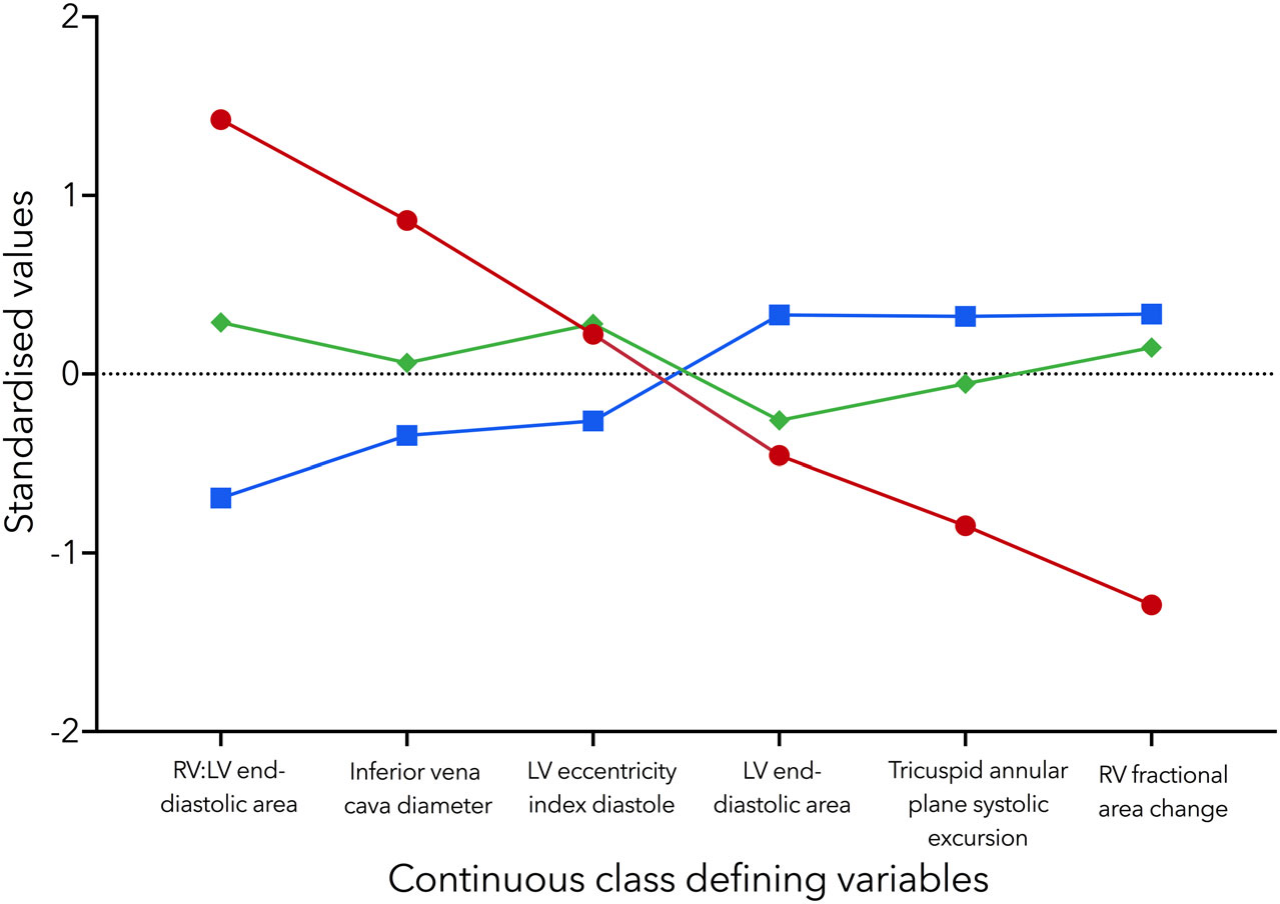
Profile plot of continuous class defining variables in the latent class analysis model. Data are plotted as their median. Class 1 (blue), Class 2 (green) and Class 3 (red). RV, right ventricular; LV, left ventricular.

All patients in class 3 had right ventricular dilation with systolic impairment, whereas patients in class 2 had mostly isolated right ventricular dilation (without systolic impairment) and patients in class 1 had mostly normal right ventricular function or isolated right ventricular systolic impairment (Fig. 2).

Ventilatory and laboratory variables were generally most deranged in class 3, least deranged in class 1 and at an intermediate level in class 2 (Table 1). Patients in classes 2 and 3 had a greater reduction in PaCO_2_ and ‘dead space fraction’ in response to prone ventilation (see online Supporting Information Figure S1) and a higher incidence of renal replacement therapy than class 1. The incidence of pulmonary embolism relative to the number of CT pulmonary angiograms performed was no different between subphenotypes (see online Supporting Information Table S1). Patients in class 3 had increased mortality compared with classes 2 and 1, respectively, whereas patients in class 2 had increased mortality compared with class 1 (p = 0.001). Subphenotype prevalence was no different in patients with echocardiography performed before or after 72 h of ICU admission (see online Supporting Information Figure S2). In 72 (23%) patients who had a second echocardiographic examination, 67 (93%) remained in the same subphenotype class. Patients who did not receive echocardiography had similar characteristics to the first subphenotype (see online Supporting Information Table S2).

## Discussion

**Figure 3:**
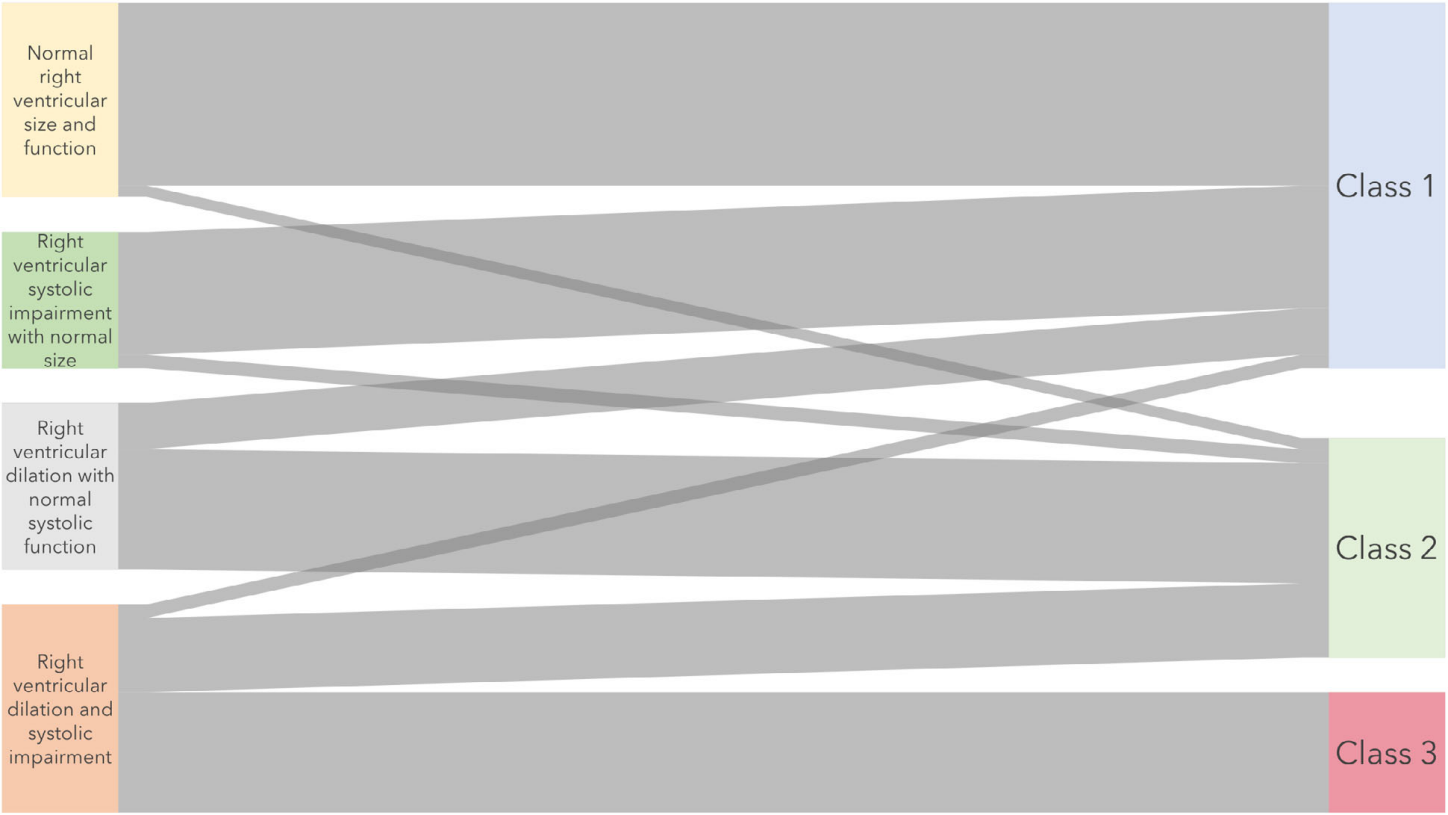
Alluvial plot demonstrating the relationship between, on the left, clinically derived subphenotypes and on the right, latent class analysis‐derived subphenotypes.

This single‐centre latent class analysis of echocardiographic and clinical variables from critically ill patients with COVID‐19 pneumonitis identified a three‐class model as the best fit for the population. Interpreting their haemodynamic profiles, we suggest the subphenotypes should be labelled as: preserved right ventricular function (class 1, 52%, mostly normal right ventricular size and function), right ventricular dysfunction (class 2, 31%, mostly dilated right ventricles with preserved systolic function) and right ventricular failure (class 3, 17%, all dilated right ventricles with severely impaired systolic function). The three subphenotypes had distinct clinical and outcome characteristics, along with differential responses to prone positioning, underscoring their potential clinical utility (Fig. 3). The subphenotypes resembled the pathophysiology of acute right ventricular failure [25, 26], but did not align clearly with current definitions of right ventricular dysfunction [27, 28], suggesting that circulatory failure mechanisms in ARDS are defined inadequately.

The characteristics of the class 2 subphenotype could be explained by the effects of the Frank‐Starling mechanism. An increased right ventricular afterload precipitates dilation, which, although associated with venous congestion and renal dysfunction [29], subsequently increases contractility and preserves stroke volume [30]. Over 90% of this subtype had normal or mildly impaired right ventricular systolic function. This maintains left ventricular filling and output, with most having normal left ventricular function and low vasopressor requirements. A risk of progression to right ventricular failure remains, conveyed by increased mortality compared with class 1 (42% vs. 22%) and whether this is mitigated by prone positioning and lung protective ventilation is an important research question. Notably, the greater reduction in ‘dead space fraction’ and PaCO_2_ with prone positioning in this subtype, with the latter better predicting survival from ARDS than the degree of oxygenation [31], suggests that prone ventilation may translate greater benefit to patients with evidence of right ventricular dysfunction.

Class 3 was characterised by greater right ventricular dilation and systolic impairment in all patients (the latter was moderate–severe in >80%). Excessive dilation can precipitate severe systolic impairment due to lengthening of sarcomeres above their optimal interactive capacity and elevated right ventricular end‐diastolic pressure decreasing the pressure gradient for coronary blood flow [32], leading to subendocardial ischaemia, which is apparent from the rise in troponin‐I levels in this subtype. Reduced right ventricular forward flow along with septal dyskinesia impairs left ventricular filling and output [33]. This is evident from reduced left ventricular size, predominantly hyperdynamic systolic function and marked rise in vasopressor requirements. This worsens right ventricular perfusion, but also blood flow to disparate organs, hastening multi‐organ dysfunction and death, with a marked increase in mortality (73%) observed in this subtype. Whether inotropic drugs, selective pulmonary vasodilators or mechanical circulatory support can improve right ventricular systolic function, mitigate harmful reductions in organ flow, abrogate this vicious cycle and improve outcomes in patients with right ventricular failure is an important research question.

However, it is unclear whether right ventricular dysfunction in ARDS contributes directly to mortality or is an epiphenomenon of disease severity, thrombotic, inflammatory or ventilatory burden. Although we found a significantly greater incidence of pulmonary embolic disease in the right ventricular failure subtype, this echocardiographic finding was often a clinical trigger for requesting pulmonary angiographic imaging and when indexed to the number of performed scans, no significant difference in the rate of pulmonary embolism between subphenotypes was found. Right ventricular dysfunction may also be precipitated by immune‐mediated processes (NETosis‐related immunothrombosis [34], monocyte recruitment and inflammasome activation [35]) that occur diffusely in the body in parallel with those occurring in the lung. In this regard, the pattern of elevated troponin, tachycardia, shock, renal dysfunction and the association with mortality common to the right ventricular failure subtype also shares notable overlap with the hyperinflammatory subphenotype described in prior non‐COVID‐19 and COVID‐19 ARDS cohorts [16]. Although our latent class analysis identified three subphenotypes as opposed to two [16], the differences may be a function of just how much organ‐ and patient‐specific data are exposed to analysis. This would not preclude the overlap of some subphenotypes and their degree of commonality warrants prospective evaluation.

The American Society of Echocardiography defines right ventricular dysfunction as right ventricular systolic impairment [27]. Surprisingly, most patients with right ventricular systolic impairment with normal sized ventricles were classified into the lowest risk of mortality – subphenotype class 1. This may be because when the right ventricular size is small, fractional area change may reflect systolic function. Right ventricular dilation (with evidence of systemic congestion) defines dysfunction in a European consensus statement [28]; however, this finding, even when coupled with systolic impairment, was unable to consistently differentiate between classes 2 and 3. This may be because right ventricular fractional area change and right:left ventricular end‐diastolic area were more severely deranged in class 3 compared with class 2 and therefore the associated thresholds that denote dysfunction may require amendment.

A novel, data‐driven, unbiased and non‐binary statistical approach was employed in this study that allowed contemporaneous assessment of multiple, complex cardiovascular parameters with low levels of missing data and intra‐ and inter‐observer variability. The classes derived are strongly separated and differentiated, stable across sensitivity analyses and ARDS duration, had pathophysiological rationale and are clinically meaningful. However, we were unable to assess more sensitive measures of right ventricular systolic impairment. Although some haemodynamic variables had to be excluded, this was expected given their co‐dependency in clinical practice and had little effect on model selection. The sample size of the study was small, although to the best of our knowledge it is the largest echocardiographic analysis in COVID‐19 pneumonitis to date. Not all eligible patients received echocardiography and therefore the findings are subject to selection bias. However, > 60% of the cohort did, with those that did not mostly resembling the preserved right ventricle subphenotype. The timing of echocardiography was not standardised, but the subphenotypes did not differ in time from symptom onset, were of similar proportion in those with early and late echocardiography and remained stable in patients who had serial echocardiography, perhaps due to the protracted course of illness. Furthermore, most examinations were performed at a time of clinical relevance, mirroring real‐world practice.

Serial examinations in larger COVID‐19 and non‐COVID‐19 ARDS cohorts at fixed time‐points across ICU admission are necessary to validate these findings and assess subphenotype stability and relationship to outcome. Prospective subphenotype derivation using parsimonious logistic regression models [36] may then allow trials of right ventricular protective measures in these predictively and prognostically enriched sub‐groups. Finally, incorporation of cardiovascular assessment with blood physiology‐based latent class analysis approaches may be desirable in order to better understand the latent structures underlying COVID‐19 and non‐COVID‐19 ARDS pathology.

## Supporting information


**Appendix** **S1.** Additional statistical analyses

**Figure S1.** Response to prone ventilation of cardiovascular subphenotypes

**Figure S2.** Comparison of cardiovascular subphenotypes in patients with early versus late transthoracic echocardiography

**Table S1.** Additional baseline characteristics and clinical parameters in cardiovascular subphenotypes.

**Table S2.** Clinical parameters in eligible patients who did not receive transthoracic echocardiography
