# Supplementary material for: Cardiovascular subphenotypes in patients with COVID‐19 pneumonitis whose lungs are mechanically ventilated: a single‐centre retrospective observational study
Source: Anaesthesia. 2022 Mar 3;77(7):763–71. doi: 10.1111/anae.15700 (PMC9314994; doi:10.1111/anae.15700)
Supplement: Supplementary file 1 — Appendix S1. Additional statistical analyses [file ANAE-77-763-s003.docx]

**Appendix S1** Additional statistical analyses

1. Exclusion of parameters prior to inclusion in the latent class analysis model

Parameters were excluded after demonstration of co-linearity prior to inclusion in the LCA model. This is demonstrated in the correlation matrix. This was expected given their co-dependency in clinical practice (e.g. mean arterial pressure and vasopressor dose, LV eccentricity index in systole vs. diastole). Due to their co-dependency, exclusion of one and inclusion of the other almost always had little effect on model selection and characteristics of the classes defined, as demonstrated below.

1.1 Correlation matrix

Continuous variables were tested for normality, and non-normal variables were transformed. Subsequently normalised variables were standardized by placement on a z-scale. The following variables were included in a correlation matrix (S-appendix figure 1): right ventricular end-diastolic area:left ventricular end-diastolic area (RVEDA:LVEDA), RVEDA, LVEDA, RV end systolic area (RVESA), LVEF = left ventricular ejection fraction, vasopressor dose, IVC = inferior vena cava diameter, c-IVC = collapsibility of IVC, TAPSE = tricuspid annular plane systolic excursion, LVEI = left ventricular eccentricity index in D (diastole) and S (systole), MAP = mean arterial pressure, HR = heart rate. Correlation between each of the variables was examined using the Pearson Correlation co-efficient and any one of two co-linear variables (correlation co-efficient >0.5) were excluded [18]. C-IVC correlated with IVC and was excluded. RVESA and RVEDA correlated with RV:LVEDA and were excluded.

Figure 1.1.1: Correlation between cardiovascular parameters

RV = right ventricular, LV = left ventricular; EDA = end-diastolic area; ESA = end-systolic area; EF = ejection fraction; IVC = inferior vena cava diameter; c-IVC = collapsibility of IVC, TAPSE = tricuspid annular plane systolic excursion; LVEI = left ventricular eccentricity index in D (diastole) and S (systole), MAP = mean arterial pressure, HR = heart rate. Values denote pearson correlation co-efficient on scale of red (-1) to blue (+1).

1.2 Sensitivity analyses

Sensitivity analyses were performed with inclusion of each of the excluded variables (C-IVC in place of IVC, RVEDA or RVESA in place of RV:LVEDA). This was performed to ascertain the effects of exclusion of these parameters on model fit / selection.

1.2.1 C-IVC

Collapsibility of IVC was included and IVC diameter was excluded. This had little effect on fit statistics: the three-class model was deemed to have the best fit due to lowest BIC, highest entropy, improvement in VLMR compared to the two class model. However the maximum bivariate residual was higher (7), which led to inclusion of IVC diameter over c-IVC.

Table 1.2.1.1 C-IVC inclusion and effect on fit statistics

| **Model** |  | **LL** | **BIC(LL)** | **AIC(LL)** | **AIC3(LL)** | **Npar** | **Max. BVR** | **VLMR** | **p-value** | **Class.Err.** | **Entropy R²** |
| --- | --- | --- | --- | --- | --- | --- | --- | --- | --- | --- | --- |
| **1** | 1-Cluster | -3213.9716 | 6519.4682 | 6459.9432 | 6475.9432 | 16 | 56.7486 |  |  | 0.0000 | 1.0000 |
| **2** | 2-Cluster | -2950.0808 | 6077.4913 | 5962.1617 | 5993.1617 | 31 | 10.9467 | 527.7816 | 0.0000 | 0.0261 | 0.8880 |
| **3** | 3-Cluster | -2877.3808 | 6017.8960 | 5846.7616 | 5892.7616 | 46 | 7.3804 | 145.4000 | 0.0000 | 0.0978 | 0.7607 |
| **4** | 4-Cluster | -2840.5213 | 6029.9816 | 5803.0426 | 5864.0426 | 61 | 5.8933 | 73.7190 | 0.0000 | 0.0949 | 0.7801 |
| **5** | 5-Cluster | -2808.9817 | 6052.7071 | 5769.9634 | 5845.9634 | 76 | 8.5397 | 63.0792 | 0.0157 | 0.1081 | 0.7753 |

LL = likelihood ratio. BIC = Bayesian information criteria. AIC = akaike information criteria. MaxBVR = maximum bivariate residual. VLMR = Vuong-Lo-Mendell-Rubin test p value

Analysis of cluster characteristics of the three-cluster model that was generated through inclusion of c-IVC demonstrated that the clusters were highly similar to the original model. Therefore the inclusion of IVC vs. C-IVC had little effect on subphenotype characterisation.

Table 1.2.1.2 Cluster characteristics with use of c-IVC

|  | **Cluster1** | **Cluster2** | **Cluster3** | **Overall** |
| --- | --- | --- | --- | --- |
| **Cluster Size** | 0.51 | 0.29 | 0.20 |  |
| **Indicators** |  |  |  |  |
| **rvlveda** |  |  |  |  |
| **Mean** | -0.666 | 0.272 | 1.319 | 0.001 |
| **rvfac** |  |  |  |  |
| **Mean** | 0.336 | 0.197 | -1.142 | -0.000 |
| **tapse** |  |  |  |  |
| **Mean** | 0.337 | -0.052 | -0.789 | -0.000 |
| **lveid** |  |  |  |  |
| **Mean** | -0.253 | 0.324 | 0.187 | 0.000 |
| **lveda** |  |  |  |  |
| **Mean** | 0.325 | -0.293 | -0.415 | -0.000 |
| **vpcat** |  |  |  |  |
| **0** | 0.549 | 0.260 | 0.152 | 0.386 |
| **1** | 0.263 | 0.282 | 0.247 | 0.265 |
| **2** | 0.1878 | 0.4567 | 0.600 | 0.347 |
| **Mean** | 0.638 | 1.196 | 1.447 | 0.960 |
| **efcat** |  |  |  |  |
| **1** | 0.113 | 0.105 | 0.081 | 0.104 |
| **2** | 0.521 | 0.513 | 0.483 | 0.518 |
| **3** | 0.364 | 0.381 | 0.435 | 0.383 |
| **Mean** | 2.251 | 2.275 | 2.353 | 2.278 |
| **civc** |  |  |  |  |
| **Mean** | 0.245 | 0.279 | -1.028 | -0.000 |

right ventricular end-diastolic area:left ventricular end-diastolic area (RV:LVEDA), LVEDA = left ventricular end-diastolic area, LVEF = left ventricular ejection fraction, vasopressor dose category, c-IVC = collapsibility of inferior vena cava diameter, TAPSE = tricuspid annular plane systolic excursion, LVEID = left ventricular eccentricity index in diastole.

- - 1. RVEDA

RVEDA was included and RV:LVEDA was excluded. Again, this had little effect on fit statistics: the three-class model was deemed to have the best fit due to a greater decrease in BIC compared to transition from cluster 3 to 4 and improvement in VLMR compared to the two class model. However, the maximum bivariate residual was higher (22), which led to inclusion of RV:LVEDA over RVEDA. Furthermore, RV:LVEDA has been used in many studies to characterise RV dysfunction.

Table 1.2.2.1 RVEDA inclusion and effect on fit statistics

| **Model** |  | **LL** | **BIC(LL)** | **AIC(LL)** | **AIC3(LL)** | **Npar** | **Max. BVR** | **VLMR** | **p-value** | **Class.Err.** | **Entropy R²** |
| --- | --- | --- | --- | --- | --- | --- | --- | --- | --- | --- | --- |
| **1** | 1-Cluster | -3213.9716 | 6519.4682 | 6459.9432 | 6475.9432 | 16 | 49.4732 |  |  | 0.0000 | 1.0000 |
| **2** | 2-Cluster | -2999.5641 | 6176.4579 | 6061.1282 | 6092.1282 | 31 | 45.8685 | 428.8150 | 0.0000 | 0.0307 | 0.8519 |
| **3** | 3-Cluster | -2918.1550 | 6099.4444 | 5928.3101 | 5974.3101 | 46 | 22.4224 | 162.8182 | 0.0000 | 0.0615 | 0.7976 |
| **4** | 4-Cluster | -2866.8244 | 6082.5877 | 5855.6487 | 5916.6487 | 61 | 12.2341 | 102.6613 | 0.0002 | 0.0998 | 0.7809 |
| **5** | 5-Cluster | -2825.4209 | 6085.5855 | 5802.8418 | 5878.8418 | 76 | 7.6491 | 82.8069 | 0.0002 | 0.1354 | 0.7695 |

LL = likelihood ratio. BIC = Bayesian information criteria. AIC = Akaike information criteria. MaxBVR = maximum bivariate residual. VLMR = Vuong-Lo-Mendell-Rubin test p value

Analysis of class characteristics of the three-class model that was generated through inclusion of RVEDA and exclusion of RV:LVEDA, demonstrated that the classes were highly similar to the original model. However cluster 3 (RV failure) was now the 2^nd^ most prevalent cluster (20%; cluster 2). The characteristics of the three clusters through comparing their mean standardised variables remained similar. Therefore the inclusion of RV:LVEDA vs. RVEDA had little effect on subphenotype characterisation. RV:LVEDA was chosen due to its use in numerous studies to characterise RV dilation.

Table 1.2.2.2 Cluster characteristics with use of RVEDA

|  | **Cluster1** | **Cluster2** | **Cluster3** | **Overall** |
| --- | --- | --- | --- | --- |
| **Cluster Size** | 0.68 | 0.19 | 0.13 |  |
| **Indicators** |  |  |  |  |
| **rvfac** |  |  |  |  |
| **Mean** | 0.323 | -1.184 | 0.073 | -0.000 |
| **tapse** |  |  |  |  |
| **Mean** | 0.222 | -0.856 | 0.111 | -0.000 |
| **lveid** |  |  |  |  |
| **Mean** | -0.136 | 0.429 | 0.075 | 0.000 |
| **lveda** |  |  |  |  |
| **Mean** | -0.082 | -0.341 | 0.962 | 0.000 |
| **vpcat** |  |  |  |  |
| **0** | 0.462 | 0.159 | 0.331 | 0.386 |
| **1** | 0.270 | 0.238 | 0.277 | 0.265 |
| **2** | 0.266 | 0.601 | 0.391 | 0.347 |
| **Mean** | 0.804 | 1.442 | 1.059 | 0.960 |
| **efcat** |  |  |  |  |
| **1** | 0.108 | 0.077 | 0.12 | 0.104 |
| **2** | 0.517 | 0.476 | 0.533 | 0.511 |
| **3** | 0.374 | 0.445 | 0.339 | 0.383 |
| **Mean** | 2.265 | 2.368 | 2.212 | 2.278 |
| **ivc** |  |  |  |  |
| **Mean** | 0.266 | -1.061 | 0.191 | -0.000 |
| **rveda** |  |  |  |  |
| **Mean** | -0.376 | 0.756 | 0.864 | 0.001 |

RVEDA = right end-diastolic area, LVEDA = left ventricular end-diastolic area, LVEF = left ventricular ejection fraction, vasopressor dose category, IVC = inferior vena cava diameter, TAPSE = tricuspid annular plane systolic excursion, LVEID = left ventricular eccentricity index in diastole.

1.2.3 RVESA

RVESA was included and RV:LVEDA was excluded. Again, this had little effect on fit statistics: the three-class model was deemed to have the best fit due to a greater decrease in BIC compared to cluster 3 to 4 and improvement in VLMR compared to the two class model. However, the maximum bivariate residual was higher (12), which led to inclusion of RV:LVEDA over RVESA. Furthermore, RV:LVEDA has been used in many studies to characterise RV dysfunction.

Table 1.2.3.1 RVESA inclusion and effect on fit statistics

| **Model** |  | **LL** | **BIC(LL)** | **AIC(LL)** | **AIC3(LL)** | **Npar** | **Max. BVR** | **VLMR** | **p-value** | **Class.Err.** | **Entropy R²** |
| --- | --- | --- | --- | --- | --- | --- | --- | --- | --- | --- | --- |
| **1** | 1-Cluster | -3213.9716 | 6519.4682 | 6459.9432 | 6475.9432 | 16 | 129.7166 |  |  | 0.0000 | 1.0000 |
| **2** | 2-Cluster | -2976.3179 | 6129.9656 | 6014.6359 | 6045.6359 | 31 | 26.6467 | 475.3074 | 0.0000 | 0.0458 | 0.8173 |
| **3** | 3-Cluster | -2919.1093 | 6101.3529 | 5930.2186 | 5976.2186 | 46 | 12.1579 | 114.4173 | 0.0001 | 0.0903 | 0.7712 |
| **4** | 4-Cluster | -2874.7757 | 6098.4904 | 5871.5514 | 5932.5514 | 61 | 9.2386 | 88.6671 | 0.0000 | 0.1064 | 0.7756 |
| **5** | 5-Cluster | -2843.6189 | 6121.9814 | 5839.2377 | 5915.2377 | 76 | 10.4916 | 62.3137 | 0.0017 | 0.1070 | 0.7928 |

LL = likelihood ratio. BIC = Bayesian information criteria. AIC = Akaike information criteria. MaxBVR = maximum bivariate residual. VLMR = Vuong-Lo-Mendell-Rubin test p value

Analysis of class characteristics of the three-class model that was generated through inclusion of RVESA and exclusion of RV:LVEDA, demonstrated that the classes were highly similar to the original model, although cluster 3 (RV failure) was now the 2^nd^ most prevalent cluster (22%; cluster 2). However the characteristics of the three clusters through comparing their mean standardised variables remained similar. Therefore the inclusion of RV:LVEDA vs. RVESA had little effect on subphenotype characterisation. RV:LVEDA was chosen due to its use in numerous studies to characterise RV dilation.

Table 1.2.3.2 Cluster characteristics with use of RVESA

|  | **Cluster1** | **Cluster2** | **Cluster3** | **Overall** |
| --- | --- | --- | --- | --- |
| **Cluster Size** | 0.58 | 0.23 | 0.19 |  |
| **Indicators** |  |  |  |  |
| **rvfac** |  |  |  |  |
| **Mean** | 0.485 | -1.259 | -0.028 | -0.000 |
| **tapse** |  |  |  |  |
| **Mean** | 0.236 | -0.782 | 0.175 | -0.000 |
| **lveid** |  |  |  |  |
| **Mean** | -0.141 | 0.289 | 0.094 | 0.000 |
| **lveda** |  |  |  |  |
| **Mean** | -0.139 | -0.276 | 0.727 | 0.000 |
| **vpcat** |  |  |  |  |
| **0** | 0.464 | 0.111 | 0.467 | 0.386 |
| **1** | 0.278 | 0.220 | 0.278 | 0.265 |
| **2** | 0.257 | 0.668 | 0.254 | 0.347 |
| **Mean** | 0.793 | 1.556 | 0.786 | 0.960 |
| **efcat** |  |  |  |  |
| **1** | 0.101 | 0.049 | 0.177 | 0.104 |
| **2** | 0.522 | 0.430 | 0.568 | 0.511 |
| **3** | 0.375 | 0.519 | 0.253 | 0.383 |
| **Mean** | 2.274 | 2.469 | 2.076 | 2.278 |
| **ivc** |  |  |  |  |
| **Mean** | -0.195 | 0.766 | -0.28 | 0.000 |
| **rvesa** |  |  |  |  |
| **Mean** | -0.602 | 1.271 | 0.363 | 0.001 |

RVESA = right end-systolic area, LVEDA = left ventricular end-diastolic area, LVEF = left ventricular ejection fraction, vasopressor dose category, IVC = inferior vena cava diameter, TAPSE = tricuspid annular plane systolic excursion, LVEID = left ventricular eccentricity index in diastole.

1. Exclusion due to local dependence after latent class analysis

Parameters were also excluded due to local dependence after LCA analysis, denoted by high bivariate residual (BVR) values generated in the fit statistics. Again, this was expected given the co-dependency of many of the parameters used in clinical practice (e.g. LVEID and septal dyskinesia, which present similar information with regards to RV function). Local dependence within classes was assessed through the use of bivariate residuals. Maximum BVR values <5 were deemed to be locally independent. The final model, after exclusion of the below parameters, had a maximum BVR of 4.98 and all class-defining variables were deemed to have conditional independence. Models with maximum BVR values >5 led to exclusion of either one of the class defining variables that were co-dependent. Sensitivity analyses were performed on excluded co-dependent variables.

2.1 LV eccentricity index in systole

When LVEIS was included alongside LVEID, the BVR amongst the two variables was 21. This led to exclusion of LVEIS. In sensitivity analyses, LVEIS was included and LVEID was excluded. Again, this had little effect on fit statistics: the three-class model was deemed to have the best fit due to lowest BIC, improvement in VLMR compared to the two class model. However, the maximum bivariate residual was higher (11), which led to inclusion of LVEID over LVEIS.

Table 2.1.1 LVEIS inclusion and effect on fit statistics

| **Model** |  | **LL** | **BIC(LL)** | **AIC(LL)** | **AIC3(LL)** | **Npar** | **Max. BVR** | **VLMR** | **p-value** | **Class.Err.** | **Entropy R²** |
| --- | --- | --- | --- | --- | --- | --- | --- | --- | --- | --- | --- |
| **1** | 1-Cluster | -3204.5543 | 6500.5810 | 6441.1086 | 6457.1086 | 16 | 57.5954 |  |  | 0.0000 | 1.0000 |
| **2** | 2-Cluster | -2995.1966 | 6167.6210 | 6052.3931 | 6083.3931 | 31 | 10.7843 | 418.7154 | 0.0000 | 0.0500 | 0.7937 |
| **3** | 3-Cluster | -2937.6228 | 6138.2289 | 5967.2456 | 6013.2456 | 46 | 7.2897 | 115.1475 | 0.0000 | 0.1006 | 0.7132 |
| **4** | 4-Cluster | -2904.5962 | 6157.9311 | 5931.1924 | 5992.1924 | 61 | 11.1038 | 66.0533 | 0.0010 | 0.0970 | 0.7489 |
| **5** | 5-Cluster | -2874.3367 | 6183.1674 | 5900.6733 | 5976.6733 | 76 | 10.6362 | 60.5191 | 0.0211 | 0.1817 | 0.6858 |

LL = likelihood ratio. BIC = Bayesian information criteria. AIC = Akaike information criteria. MaxBVR = maximum bivariate residual. VLMR = Vuong-Lo-Mendell-Rubin test p value

Analysis of class characteristics of the three-class model that was generated through inclusion of LVEIS and exclusion of LVEID, demonstrated that the classes were highly similar to the original model. Therefore the inclusion of LVEID vs. LVEIS had little effect on subphenotype characterisation. LVEID was chosen due to the lower BVR when it was included in the model.

Table 2.1.2 Cluster characteristics with use of LVEIS

|  | **Cluster1** | **Cluster2** | **Cluster3** | **Overall** |
| --- | --- | --- | --- | --- |
| **Cluster Size** | 0.61 | 0.20 | 0.19 |  |
| **Indicators** |  |  |  |  |
| **rvfac** |  |  |  |  |
| **Mean** | 0.422 | 0.030 | -1.271 | 0.002 |
| **tapse** |  |  |  |  |
| **Mean** | 0.264 | 0.004 | -0.806 | -0.003 |
| **lveda** |  |  |  |  |
| **Mean** | 0.072 | 0.275 | -0.499 | -0.000 |
| **vpcat** |  |  |  |  |
| **0** | 0.473 | 0.426 | 0.096 | 0.388 |
| **1** | 0.278 | 0.285 | 0.210 | 0.266 |
| **2** | 0.248 | 0.288 | 0.692 | 0.345 |
| **Mean** | 0.774 | 0.861 | 1.596 | 0.957 |
| **efcat** |  |  |  |  |
| **1** | 0.117 | 0.111 | 0.061 | 0.105 |
| **2** | 0.530 | 0.524 | 0.450 | 0.513 |
| **3** | 0.351 | 0.363 | 0.487 | 0.381 |
| **Mean** | 2.234 | 2.252 | 2.426 | 2.276 |
| **ivc** |  |  |  |  |
| **Mean** | -0.233 | -0.117 | 0.803 | -0.001 |
| **rvlveda** |  |  |  |  |
| **Mean** | -0.393 | -0.166 | 1.355 | 0.003 |
| **lveis** |  |  |  |  |
| **Mean** | -0.291 | 0.324 | 0.539 | 0.000 |

RVESA = right end-systolic area, LVEDA = left ventricular end-diastolic area, LVEF = left ventricular ejection fraction, vasopressor dose category, IVC = inferior vena cava diameter, TAPSE = tricuspid annular plane systolic excursion, LVEIS = left ventricular eccentricity index in systole.

- 1. Mean arterial pressure

When MAP was included alongside VP category, the BVR amongst the two variables was 22. This led to exclusion of MAP. In sensitivity analyses, MAP was included and VP category was excluded. Again, this had little effect on fit statistics: the three-class model was deemed to have the best fit due to lowest BIC, improvement in VLMR compared to the two class model. However, the maximum bivariate residual was higher (6.5), which led to inclusion of VP category over MAP.

Table 2.2.1 MAP inclusion and effect on fit statistics

| **Model** |  | **LL** | **BIC(LL)** | **AIC(LL)** | **AIC3(LL)** | **Npar** | **Max. BVR** | **VLMR** | **p-value** | **Class.Err.** | **Entropy R²** |
| --- | --- | --- | --- | --- | --- | --- | --- | --- | --- | --- | --- |
| **1** | 1-Cluster | -3314.7677 | 6721.0603 | 6661.5353 | 6677.5353 | 16 | 56.7486 |  |  | 0.0000 | 1.0000 |
| **2** | 2-Cluster | -3103.0342 | 6389.1184 | 6270.0684 | 6302.0684 | 32 | 6.5524 | 423.4669 | 0.0000 | 0.0370 | 0.8206 |
| **3** | 3-Cluster | -3047.9095 | 6370.3940 | 6191.8190 | 6239.8190 | 48 | 6.5010 | 110.2494 | 0.0000 | 0.1208 | 0.7149 |
| **4** | 4-Cluster | -3002.6827 | 6371.4654 | 6133.3654 | 6197.3654 | 64 | 8.1036 | 90.4536 | 0.0000 | 0.1540 | 0.7017 |
| **5** | 5-Cluster | -2972.6399 | 6402.9047 | 6105.2797 | 6185.2797 | 80 | 6.1284 | 60.0857 | 0.0010 | 0.1523 | 0.7258 |

LL = likelihood ratio. BIC = Bayesian information criteria. AIC = Akaike information criteria. MaxBVR = maximum bivariate residual. VLMR = Vuong-Lo-Mendell-Rubin test p value

Analysis of class characteristics of the three-class model that was generated through inclusion of MAP and exclusion of VP category, demonstrated that the classes were highly similar to the original model. Therefore the inclusion of MAP vs. VP category had little effect on subphenotype characterisation. VP Category was chosen due to the lower BVR and because MAP targets can be 65mmHg in ICU patients but vasopressor requirements can vary considerably. VP category was therefore deemed to be more sensitive at describing the degree of shock compared to MAP.

Table 2.2.2 Cluster characteristics with use of MAP

|  | **Cluster1** | **Cluster2** | **Cluster3** | **Overall** |
| --- | --- | --- | --- | --- |
| Cluster Size | 0.48 | 0.35 | 0.17 |  |
| Indicators |  |  |  |  |
| rvfac |  |  |  |  |
| Mean | 0.348 | 0.146 | -1.279 | -0.000 |
| tapse |  |  |  |  |
| Mean | 0.361 | -0.068 | -0.875 | -0.000 |
| lveda |  |  |  |  |
| Mean | 0.345 | -0.244 | -0.470 | -0.000 |
| efcat |  |  |  |  |
| 1 | 0.108 | 0.119 | 0.065 | 0.104 |
| 2 | 0.518 | 0.528 | 0.456 | 0.511 |
| 3 | 0.372 | 0.352 | 0.478 | 0.38 |
| Mean | 2.264 | 2.233 | 2.412 | 2.278 |
| ivc |  |  |  |  |
| Mean | -0.352 | 0.061 | 0.865 | 0.000 |
| rvlveda |  |  |  |  |
| Mean | -0.690 | 0.253 | 1.424 | 0.001 |
| lveid |  |  |  |  |
| Mean | -0.260 | 0.257 | 0.206 | 0.000 |
| map |  |  |  |  |
| Mean | 0.237 | -0.122 | -0.416 | -0.000 |

RVESA = right end-systolic area, LVEDA = left ventricular end-diastolic area, LVEF = left ventricular ejection fraction, vasopressor dose category, IVC = inferior vena cava diameter, TAPSE = tricuspid annular plane systolic excursion, LVEID = left ventricular eccentricity index in diastole. MAP = mean arterial pressure.

2.3 Tricuspid regurgitation

When TR was included alongside LVEF, the BVR amongst the two variables was 25. This led to exclusion of TR. In sensitivity analyses, TR was included and LVEF removed. The three-class model was deemed to have the best fit. This was because the cluster 2 to 3 had a greater decrease in BIC and AIC compared to the transition from cluster 3 to 4. However, the maximum bivariate residual was higher (49), which led to inclusion of LVEF over TR.

Table 2.3.1 TR inclusion and effect on fit statistics

| **Model** |  | **LL** | **BIC(LL)** | **AIC(LL)** | **AIC3(LL)** | **Npar** | **Max. BVR** | **VLMR** | **p-value** | **Class.Err.** | **Entropy R²** |
| --- | --- | --- | --- | --- | --- | --- | --- | --- | --- | --- | --- |
| **1** | 1-Cluster | -3746.5075 | 7597.2342 | 7529.0150 | 7547.0150 | 18 | 397.4035 |  |  | 0.0000 | 1.0000 |
| **2** | 2-Cluster | -3571.9085 | 7340.6756 | 7211.8170 | 7245.8170 | 34 | 393.7058 | 349.1980 | 0.0000 | 0.0416 | 0.8143 |
| **3** | 3-Cluster | -3449.9575 | 7189.4131 | 6999.9150 | 7049.9150 | 50 | 49.3142 | 243.9019 | 0.0000 | 0.0199 | 0.9339 |
| **4** | 4-Cluster | -3345.7706 | 7073.6785 | 6823.5411 | 6889.5411 | 66 | 8.1072 | 208.3739 | 0.0000 | 0.0261 | 0.9435 |
| **5** | 5-Cluster | -3287.6406 | 7050.0580 | 6739.2813 | 6821.2813 | 82 | 4.4498 | 116.2598 | 0.0000 | 0.0646 | 0.8985 |

LL = likelihood ratio. BIC = Bayesian information criteria. AIC = Akaike information criteria. MaxBVR = maximum bivariate residual. VLMR = Vuong-Lo-Mendell-Rubin test p value

Analysis of class characteristics of the three-class model that was generated through inclusion of TR and exclusion of LVEF, demonstrated that the classes were different to the original model. The presence of a severe RV failure cluster (cluster 3) was still evident, but was smaller and predominantly defined by moderate-severe TR. Cluster 1 and 2 were different. This may be because of exclusion of LVEF which was influential in deriving the previous clusters. Effect of RV function on LV function (as assessed by LVEF) was deemed more useful for inclusion in a cardiovascular model than presence of TR and was therefore chosen in the model.

Table 2.3.2 Cluster characteristics with use of TR

|  | **Cluster1** | **Cluster2** | **Cluster3** | **Overall** |
| --- | --- | --- | --- | --- |
| Cluster Size | 0.61 | 0.27 | 0.12 |  |
| Indicators |  |  |  |  |
| RVFAC |  |  |  |  |
| Mean | 0.098 | 0.322 | -1.193 | -0.000 |
| LVEID |  |  |  |  |
| Mean | -0.083 | -0.256 | 0.973 | 0.000 |
| ivc |  |  |  |  |
| Mean | 0.072 | -0.489 | 0.700 | 0.000 |
| lveda |  |  |  |  |
| Mean | 0.075 | 0.191 | -0.795 | -0.000 |
| tapse |  |  |  |  |
| Mean | -0.028 | 0.394 | -0.715 | -0.000 |
| vpcat |  |  |  |  |
| 1 | 0.431 | 0.415 | 0.089 | 0.385 |
| 2 | 0.263 | 0.264 | 0.184 | 0.253 |
| 3 | 0.305 | 0.320 | 0.725 | 0.360 |
| Mean | 0.436 | 0.452 | 0.818 | 0.487 |
| rvlveda |  |  |  |  |
| Mean | -0.033 | -0.549 | 1.360 | 0.000 |
| tr |  |  |  |  |
| 1 | 0.160 | 0.000 | 0.000 | 0.097 |
| 2 | 0.783 | 0.083 | 0.054 | 0.507 |
| 3 | 0.056 | 0.916 | 0.945 | 0.394 |
| Mean | 0.448 | 0.958 | 0.972 | 0.648 |

RVESA = right end-systolic area, LVEDA = left ventricular end-diastolic area, TR = tricuspid regurgitation, vasopressor dose category, IVC = inferior vena cava diameter, TAPSE = tricuspid annular plane systolic excursion, LVEID = left ventricular eccentricity index in diastole.

2.4 Heart rate

When HR was included alongside vasopressor category, the BVR amongst the two variables was 20. This led to exclusion of HR. In sensitivity analyses, HR was included and vasopressor category was excluded. Again, this had little effect on fit statistics: the three-class model was deemed to have the best fit due to lowest BIC, improvement in VLMR compared to the two-class model. However, the maximum bivariate residual was higher (5.6), which led to inclusion of Vasopressor category over HR.

Table 2.4.1 HR inclusion and effect on fit statistics

| **Model** |  | **LL** | **BIC(LL)** | **AIC(LL)** | **AIC3(LL)** | **Npar** | **Max. BVR** | **VLMR** | **p-value** | **Class.Err.** | **Entropy R²** |
| --- | --- | --- | --- | --- | --- | --- | --- | --- | --- | --- | --- |
| **1** | 1-Cluster | -3357.4074 | 6806.3398 | 6746.8148 | 6762.8148 | 16 | 56.7486 |  |  | 0.0000 | 1.0000 |
| **2** | 2-Cluster | -3129.4342 | 6441.9183 | 6322.8684 | 6354.8684 | 32 | 10.2771 | 455.9464 | 0.0000 | 0.0467 | 0.8137 |
| **3** | 3-Cluster | -3078.0036 | 6430.5822 | 6252.0072 | 6300.0072 | 48 | 5.6817 | 102.8611 | 0.0000 | 0.1050 | 0.7462 |
| **4** | 4-Cluster | -3036.4596 | 6439.0191 | 6200.9192 | 6264.9192 | 64 | 3.2076 | 83.0881 | 0.0031 | 0.1282 | 0.7406 |
| **5** | 5-Cluster | -3008.7586 | 6475.1422 | 6177.5173 | 6257.5173 | 80 | 2.7537 | 55.4019 | 0.0057 | 0.1677 | 0.7199 |

LL = likelihood ratio. BIC = Bayesian information criteria. AIC = Akaike information criteria. MaxBVR = maximum bivariate residual. VLMR = Vuong-Lo-Mendell-Rubin test p value

Analysis of class characteristics of the three-class model that was generated through inclusion of HR and exclusion of vasopressor category, demonstrated that the classes were highly similar to the original model. Therefore the inclusion of HR vs. VP category had little effect on subphenotype characterisation. VP category was chosen due to the lower BVR and its increased utility in characterising cardiovascular function and degree of shock compared to HR.

Table 2.4.2 Cluster characteristics with use of HR

|  | Cluster1 | Cluster2 | Cluster3 | Overall |
| --- | --- | --- | --- | --- |
| Cluster Size | 0.51 | 0.32 | 0.17 |  |
| Indicators |  |  |  |  |
| rvfac |  |  |  |  |
| Mean | 0.335 | 0.147 | -1.290 | -0.000 |
| tapse |  |  |  |  |
| Mean | 0.322 | -0.049 | -0.882 | -0.000 |
| lveda |  |  |  |  |
| Mean | 0.323 | -0.282 | -0.447 | -0.000 |
| ivc |  |  |  |  |
| Mean | -0.326 | 0.060 | 0.871 | 0.000 |
| rvlveda |  |  |  |  |
| Mean | -0.677 | 0.317 | 1.453 | 0.001 |
| lveid |  |  |  |  |
| Mean | -0.256 | 0.298 | 0.217 | 0.000 |
| hr |  |  |  |  |
| Mean | -0.105 | 0.101 | 0.127 | 0.000 |
| vpcat |  |  |  |  |
| 0 | 0.557 | 0.253 | 0.122 | 0.386 |
| 1 | 0.263 | 0.285 | 0.234 | 0.265 |
| 2 | 0.179 | 0.461 | 0.642 | 0.347 |
| Mean | 0.621 | 1.207 | 1.519 | 0.960 |

RVESA = right end-systolic area, LVEDA = left ventricular end-diastolic area, LVEF = left ventricular ejection fraction, IVC = inferior vena cava diameter, TAPSE = tricuspid annular plane systolic excursion, LVEID = left ventricular eccentricity index in diastole. HR = heart rate.

2.5 Septal dyskinesia

When septal dyskinesia was included alongside LVEID, the BVR amongst the two variables was 29. This led to exclusion of septal dyskinesia. In sensitivity analyses, septal dyskinesia was included and LVEID was excluded. Again, this had little effect on fit statistics: the three-class model was deemed to have the best fit due to lowest BIC, improvement in VLMR compared to the two class model. However, the maximum bivariate residual was higher (8), which led to inclusion of LVEID over septal dyskinesia.

Table 2.5.1 Septal dyskinesia inclusion and effect on fit statistics

| **Model** |  | **LL** | **BIC(LL)** | **AIC(LL)** | **AIC3(LL)** | **Npar** | **Max. BVR** | **VLMR** | **p-value** | **Class.Err.** | **Entropy R²** |
| --- | --- | --- | --- | --- | --- | --- | --- | --- | --- | --- | --- |
| **1** | 1-Cluster | -3688.8565 | 7481.9323 | 7413.7131 | 7431.7131 | 18 | 44.5817 |  |  | 0.0000 | 1.0000 |
| **2** | 2-Cluster | -3506.3522 | 7215.3530 | 7082.7044 | 7117.7044 | 35 | 9.7517 | 365.0086 | 0.0000 | 0.0381 | 0.8228 |
| **3** | 3-Cluster | -3457.0631 | 7205.2042 | 7018.1263 | 7070.1263 | 52 | 7.7929 | 98.5781 | 0.0000 | 0.1373 | 0.6821 |
| **4** | 4-Cluster | -3425.5656 | 7250.6385 | 6989.1313 | 7058.1313 | 69 | 8.7011 | 62.9950 | 0.0009 | 0.1301 | 0.7172 |
| **5** | 5-Cluster | -3394.1665 | 7286.2696 | 6960.3330 | 7046.3330 | 86 | 7.9231 | 62.7983 | 0.0327 | 0.1344 | 0.7484 |

LL = likelihood ratio. BIC = Bayesian information criteria. AIC = Akaike information criteria. MaxBVR = maximum bivariate residual. VLMR = Vuong-Lo-Mendell-Rubin test p value

Analysis of class characteristics of the three-class model that was generated through inclusion of septal dyskinesia and exclusion of LVEID, demonstrated that the classes were highly similar to the original model. Therefore the inclusion of LVEID vs. septal dyskinesia had little effect on subphenotype characterisation. LVEID was chosen due to the lower BVR and its quantitative assessment of RV volume overload, as opposed to qualititative and therefore subjective assessment of septal dyskinesia.

Table 2.5.2 Cluster characteristics with use of septal dyskinesia

|  | **Cluster1** | **Cluster2** | **Cluster3** | **Overall** |
| --- | --- | --- | --- | --- |
| Cluster Size | 0.48 | 0.36 | 0.16 |  |
| Indicators |  |  |  |  |
| Efcat |  |  |  |  |
| 1 | 0.077 | 0.133 | 0.061 | 0.094 |
| 2 | 0.503 | 0.563 | 0.473 | 0.519 |
| 3 | 0.419 | 0.303 | 0.464 | 0.385 |
| Mean | 2.342 | 2.169 | 2.403 | 2.290 |
| RVFAC |  |  |  |  |
| Mean | 0.331 | 0.174 | -1.399 | -0.000 |
| Ivc |  |  |  |  |
| Mean | -0.351 | 0.057 | 0.936 | 0.000 |
| Lveda |  |  |  |  |
| Mean | 0.329 | -0.236 | -0.467 | -0.000 |
| Tapse |  |  |  |  |
| Mean | 0.389 | -0.133 | -0.881 | -0.000 |
| vpcat |  |  |  |  |
| 1 | 0.544 | 0.301 | 0.094 | 0.385 |
| 2 | 0.255 | 0.275 | 0.199 | 0.253 |
| 3 | 0.200 | 0.422 | 0.706 | 0.360 |
| Mean | 0.328 | 0.560 | 0.805 | 0.487 |
| rvlveda |  |  |  |  |
| Mean | -0.606 | 0.182 | 1.426 | 0.001 |
| septal |  |  |  |  |
| 0 | 0.239 | 0.28 | 0.125 | 0.238 |
| 1 | 0.750 | 0.648 | 0.666 | 0.700 |
| 2 | 0.010 | 0.064 | 0.208 | 0.061 |

RVESA = right end-systolic area, LVEDA = left ventricular end-diastolic area, LVEF = left ventricular ejection fraction, vasopressor dose category, IVC = inferior vena cava diameter, TAPSE = tricuspid annular plane systolic excursion, septal dyskinesia.

1. Reasons for choosing 3 cluster model

3.1 Rejection of two cluster model

BIC decreased from classes 1 – 3, but increased when classes 4 / 5 were added. AIC decreased sequentially, however the rate of decrease was less with the addition of classes 4 / 5. Compared to the two-class model, the three-class model had an improved model fit (VLMR = p<0.0001) and higher entropy indicating good class separation and was therefore judged to be the best fit for the population.

- 1. Rejection of Four cluster model

Whilst the VLMR test demonstrated an improved model fit with the addition of a fourth class (p=0.011), the increase in BIC and decreased reduction in AIC with the addition of a fourth class resulted in rejection of this model.

Table 3.2.1: Cluster characteristics of four cluster model

|  | Cluster1 | Cluster2 | Cluster3 | Cluster4 | Overall |
| --- | --- | --- | --- | --- | --- |
| Cluster Size | 0.44 | 0.26 | 0.18 | 0.12 |  |
| Indicators |  |  |  |  |  |
| rvlveda |  |  |  |  |  |
| Mean | -0.727 | 0.068 | 1.387 | 0.510 | 0.001 |
| rvfac |  |  |  |  |  |
| Mean | 0.312 | 0.408 | -1.363 | -0.041 | -0.000 |
| tapse |  |  |  |  |  |
| Mean | 0.346 | 0.008 | -0.855 | -0.048 | -0.000 |
| ivc |  |  |  |  |  |
| Mean | -0.426 | 0.243 | 0.798 | -0.109 | 0.000 |
| lveid |  |  |  |  |  |
| Mean | -0.295 | 0.157 | 0.426 | 0.131 | 0.000 |
| lveda |  |  |  |  |  |
| Mean | 0.372 | -0.299 | -0.501 | 0.000 | -0.000 |
| vpcat |  |  |  |  |  |
| 0 | 0.585 | 0.261 | 0.083 | 0.365 | 0.386 |
| 1 | 0.260 | 0.295 | 0.210 | 0.302 | 0.265 |
| 2 | 0.153 | 0.443 | 0.706 | 0.332 | 0.347 |
| Mean | 0.568 | 1.181 | 1.622 | 0.966 | 0.960 |
| efcat |  |  |  |  |  |
| 1 | 0.111 | 0.114 | 0.053 | 0.137 | 0.104 |
| 2 | 0.524 | 0.527 | 0.432 | 0.544 | 0.511 |
| 3 | 0.364 | 0.358 | 0.513 | 0.318 | 0.383 |
| Mean | 2.253 | 2.244 | 2.460 | 2.181 | 2.278 |

RVESA = right end-systolic area, LVEDA = left ventricular end-diastolic area, LVEF = left ventricular ejection fraction, vasopressor dose category, IVC = inferior vena cava diameter, TAPSE = tricuspid annular plane systolic excursion, LVEID = left ventricular eccentricity index in diastole.

1. Assessment of subphenotype stability

A limitation of the study is that the timing of TTE after ICU admission was not standardised. This may have had an effect on subphenotype classification as this occurred at different time points amongst patients. To assess whether the timing of TTE affected the derivation of the subphenotype class, we compared subphenotype classification in patients with two TTE’s. We compared the classification of their 1^st^ TTE to their 2^nd^. If subphenotype derivation was similar between the two TTE’s, then it provides evidence of subphenotype stability and that the timing of TTE had little influence on the class of subphenotype derived

4.1 Analysis of second TTE examinations

Seventy-two patients (23.6%) had a 2^nd^ TTE performed at a median time of day 15 (10 – 19) whilst being invasively ventilated in the ICU. Whilst the parameters from the 1^st^ TTE was included in all prior analyses, in this analysis, for the 72 patients with 2 TTE’s, data from the 2^nd^ TTE was included instead. The remaining patients with only one TTE examination were included as before. The latent class analysis was performed again and the results are demonstrated below (Table 4.1.1). Inclusion of haemodynamic parameters from the 2^nd^ TTE rather than the 1^st^ had little effect on fit statistics: the three-class model was deemed to have the best fit due to lowest BIC, improvement in VLMR compared to the two class model.

Table 4.1.1 Fit statistics for one to five class models of latent class analysis using 2^nd^ TTE data

| **Model** |  | **LL** | **BIC(LL)** | **AIC(LL)** | **AIC3(LL)** | **Npar** | **Max. BVR** | **VLMR** | **p-value** | **Class.Err.** | **Entropy R²** |
| --- | --- | --- | --- | --- | --- | --- | --- | --- | --- | --- | --- |
| **1** | 1-Cluster | -3182.5177 | 6456.4022 | 6397.0353 | 6413.0353 | 16 | 56.5298 |  |  | 0.0000 | 1.0000 |
| **2** | 2-Cluster | -2998.3711 | 6173.7654 | 6058.7421 | 6089.7421 | 31 | 7.1783 | 368.2932 | 0.0000 | 0.0401 | 0.8213 |
| **3** | 3-Cluster | -2951.4357 | 6165.5511 | 5994.8715 | 6040.8715 | 46 | 7.8531 | 93.8707 | 0.0000 | 0.1391 | 0.7919 |
| **4** | 4-Cluster | -2925.3551 | 6199.0463 | 5972.7102 | 6033.7102 | 61 | 6.3439 | 52.1612 | 0.0477 | 0.1533 | 0.7028 |
| **5** | 5-Cluster | -2896.0496 | 6226.0916 | 5944.0991 | 6020.0991 | 76 | 6.1025 | 58.6111 | 0.0066 | 0.1519 | 0.7294 |

LL = likelihood ratio. BIC = Bayesian information criteria. AIC = Akaike information criteria. MaxBVR = maximum bivariate residual. VLMR = Vuong-Lo-Mendell-Rubin test p value

Analysis of class characteristics of the three-class model generated through use of 2^nd^ TTE data demonstrated that the classes were highly similar to the 1^st^ TTE data model.

Table 4.1.2 Cluster characteristics when using 2^nd^ TTE data

|  | **Cluster1** | **Cluster2** | **Cluster3** | **Overall** |
| --- | --- | --- | --- | --- |
| Cluster Size | 0.43 | 0.38 | 0.19 |  |
| Indicators |  |  |  |  |
| EF |  |  |  |  |
| 1 | 0.102 | 0.124 | 0.060 | 0.102 |
| 2 | 0.515 | 0.535 | 0.449 | 0.509 |
| 3 | 0.382 | 0.340 | 0.490 | 0.387 |
| Mean | 2.279 | 2.215 | 2.429 | 2.284 |
| vpcat |  |  |  |  |
| 0 | 0.546 | 0.312 | 0.111 | 0.374 |
| 1 | 0.260 | 0.284 | 0.219 | 0.261 |
| 2 | 0.193 | 0.402 | 0.669 | 0.364 |
| Mean | 0.646 | 1.090 | 1.558 | 0.990 |
| TAPSE |  |  |  |  |
| Mean | 0.393 | -0.025 | -0.839 | -0.003 |
| ivc |  |  |  |  |
| Mean | -0.373 | -0.005 | 0.831 | -0.001 |
| lveid |  |  |  |  |
| Mean | -0.382 | 0.235 | 0.410 | 0.003 |
| lveda |  |  |  |  |
| Mean | 0.401 | -0.236 | -0.473 | -0.007 |
| rvlveda |  |  |  |  |
| Mean | -0.663 | 0.063 | 1.369 | 0.003 |
| rvfac |  |  |  |  |
| Mean | 0.384 | 0.233 | -1.304 | 0.000 |

Individuals were allocated to their most likely class based on a posterior probability of class assignment >50%. Comparison of class assignment in patients 1^st^ TTE to their 2^nd^ is outlined in Figure 4.1.3. Patients remained in the same class in 93.1% (67/72).

Figure 4.1.1 Subphenotype stability in patients with 2 TTE examinations

Comparison of 1^st^ TTE subphenotype classification (left) to 2^nd^ TTE subphenotype classification in patients with 2 TTE examinations (n=72)

Altogether, this suggests that patients remained in the same subphenotype during their period of invasive ventilation for COVID-19 ARDS. This implies that the timing of TTE (early or late) had little influence on the subphenotype derived for each patient.
