# Supplementary figures and images for: Cardiovascular subphenotypes in patients with COVID‐19 pneumonitis whose lungs are mechanically ventilated: a single‐centre retrospective observational study
Source: Anaesthesia. 2022 Mar 3;77(7):763–71. doi: 10.1111/anae.15700 (PMC9314994; doi:10.1111/anae.15700)

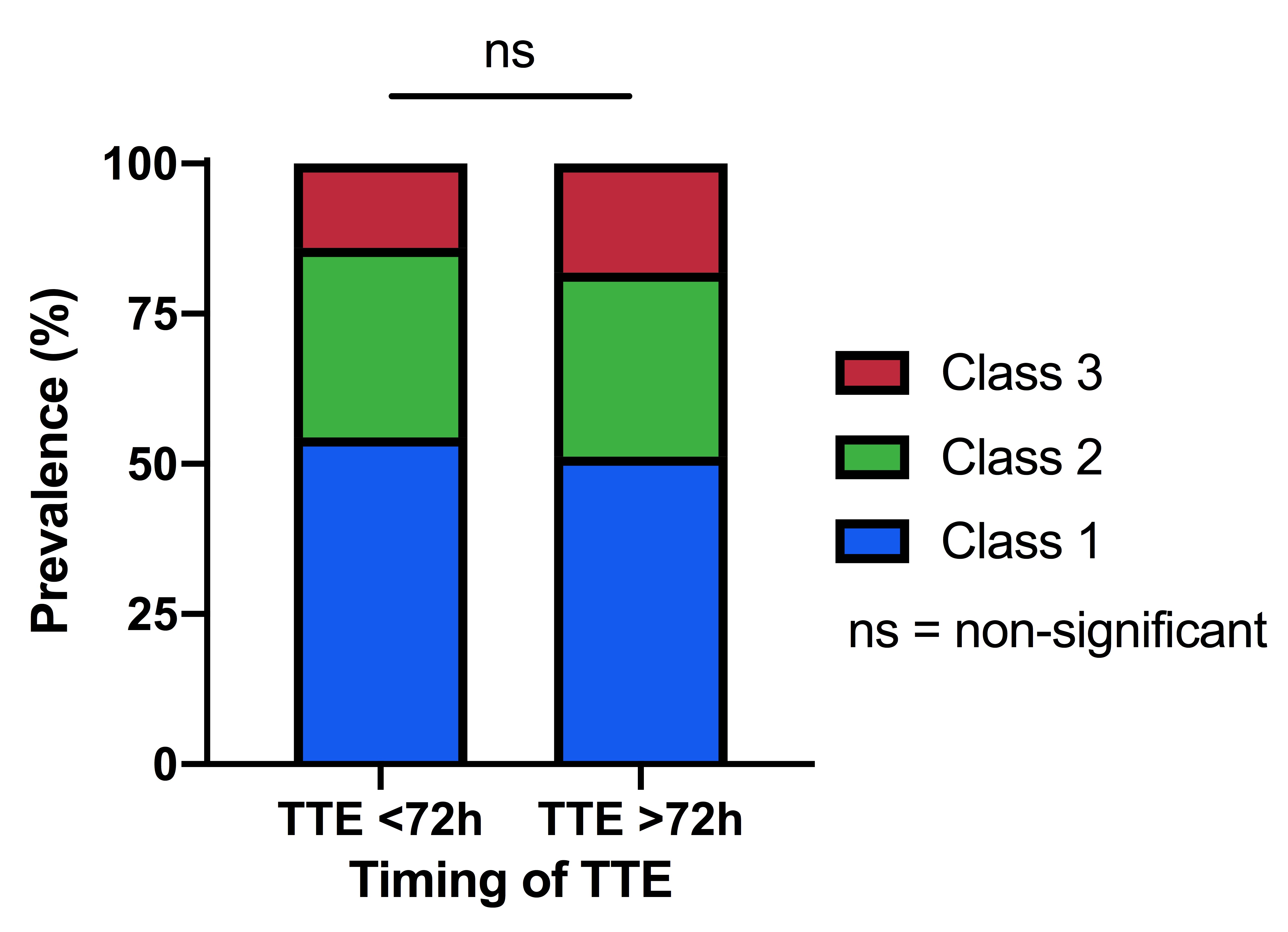

Supplement: Supplementary file 3 — Figure S2. Comparison of cardiovascular subphenotypes in patients with early versus late transthoracic echocardiography [file ANAE-77-763-s002.jpg]
