# Supplementary material for: Cardiovascular subphenotypes in patients with COVID‐19 pneumonitis whose lungs are mechanically ventilated: a single‐centre retrospective observational study
Source: Anaesthesia. 2022 Mar 3;77(7):763–71. doi: 10.1111/anae.15700 (PMC9314994; doi:10.1111/anae.15700)
Supplement: Supplementary file 4 — Table S1. Additional baseline characteristics and clinical parameters in cardiovascular subphenotypes. [file ANAE-77-763-s005.docx]

**Table S1**: Additional patient characteristics and clinical parameters in cardiovascular subphenotypes. Values are number (proportion) or median (IQR).

|  | All N=305 | Class 1  N=158 | Class 2  N=95 | Class 3  N=52 | P value |
| --- | --- | --- | --- | --- | --- |
| **Characteristics** |  |  |  |  |  |
| Clinical Frailty score | 2 (1–2) | 2 (1–2) | 2 (2–2) | 2 (1–3) | 0.657 |
| Comorbidities |  |  |  |  | 0.577 |
| Diabetes mellitus | 94 (30.8) | 44 (27.9) | 31 (32.6) | 19 (36.5) |  |
| Obesity | 131 (43.0) | 75 (47.5) | 39 (41.1) | 17 (32.7) |  |
| Hypertension | 107 (35.1) | 53 (33.5) | 40 (42.1) | 14 (26.9) |  |
| Ischaemic heart disease | 26 (8.5) | 16 (10.1) | 6 (6.3) | 4 (7.7) |  |
| Chronic obstructive pulmonary disease / Asthma | 48 (15.7) | 23 (14.6) | 17 (17.9) | 8 (15.4) |  |
| Chronic kidney disease | 34 (11.1) | 18 (11.4) | 14 (14.7) | 2 (3.9) |  |
| None | 91 (29.8) | 49 (31.0) | 25 (26.3) | 17 (32.7) |  |
| Ethnicity |  |  |  |  | 0.384 |
| White | 158 (51.8) | 79 (50.0) | 45 (47.4) | 34 (65.4) |  |
| Asian | 111 (36.4) | 59 (37.3) | 39 (41.1) | 13 (25.0) |  |
| Black | 22 (7.2) | 12 (7.6) | 6 (6.3) | 4 (7.7) |  |
| Mixed race | 4 (1.3) | 1 (0.6) | 3 (3.2) | 0 |  |
| Other | 10 (3.3) | 7 (4.4) | 2 (2.1) | 1 (1.9) |  |
| Day from symptom onset to echocardiogram | 18 (12 – 26) | 18 (12 – 26) | 19 (11 – 29) | 18 (11 – 25) | 0.590 |
| Day from hospital admission to echocardiogram | 13 (9 – 19) | 13 (9 – 19) | 14 (8 – 18) | 11 (6 – 16) | 0.482 |
| **Clinical variables** |  |  |  |  |  |
| Mean tidal volume; mls/kg/PBW | 7.2 (6.7 – 7.6) | 7.2 (6.8 – 7.6) | 7.2 (6.5 – 7.6) | 7.1 (6.7 – 7.6) | 0.995 |
| Percentage time spent between 6-8mls/kg PBW | 65 (53 – 75%) | 66 (53 – 75) | 65 (48 – 74) | 69 (59 – 78) | 0.577 |
| Cumulative fluid balance; mLs | -342 (-964 – +326) | -345 (-1011 – +456) | -381 (-1008 – +229) | -121 (-747 – +334) | 0.337 |
| Ventilation rate; bpm | 20 (19 – 22) | 20 (18 – 22) | 20 (20 – 22) | 20 (20 – 23) | 0.075 |
| Alanine transaminase; IU L^-1^; n=294) | 37 (24 – 72) | 36 (23 – 64) | 34 (23 – 72) | 41 (26 – 111) | 0.328 |
| Alkaline phosphatase; IU L^-1^; n=294) | 93 (67 – 131) | 92 (69 – 126) | 93 (69 – 143) | 95 (60 – 131) | 0.388 |
| Creatinine mg/dL | 82 (59 – 150) | 73 (55 – 119) | 86 (63 – 145) | 108 (60 – 190) | 0.021 |
| Computerised tomography pulmonary angiogram | 111 (36.4) | 59 (37.3) | 28 (29.5) | 24 (46.2) | 0.125 |
| Pulmonary embolism diagnosed | 29 (9.5) | 11 (7.0) | 8 (8.4) | 10 (19.2) | 0.030 |
| Pulmonary embolism relative to computerised tomography pulmonary angiogram | 29 (26.1) | 11 (18.6) | 8 (28.6) | 10 (41.7) | 0.091 |
| **Echocardiographic parameters** |  |  |  |  |  |
| Right ventricular systolic impairment severity |  |  |  |  | <0.001 |
| Mild (25 – 35) | 89 (29.2) | 50 (31.7) | 29 (30.5) | 10 (19.2) |  |
| Moderate (18 – 24.9) | 47 (15.4) | 10 (6.3) | 7 (7.3) | 30 (57.7) |  |
| Severe (<18) | 13 (4.3) | 0 | 1 (1.1) | 12 (23.1) |  |
| Right ventricular dilation severity |  |  |  |  | <0.001 |
| Mild (0.6 – 0.8) | 90 (29.5) | 23 (14.6) | 60 (63.2) | 7 (13.5) |  |
| Moderate (0.8 – 1.0) | 56 (18.4) | 0 | 23 (24.2) | 33 (63.5) |  |
| Severe (≥1.0) | 12 (3.9) | 0 | 0 | 12 (23.1) |  |
| Tricuspid regurgitation |  |  |  |  | <0.001 |
| None | 81 (26.6) | 43 (27.2) | 30 (31.6) | 8 (15.4) |  |
| Mild | 203 (66.6) | 111 (70.3) | 60 (63.2) | 32 (61.5) |  |
| Moderate | 20 (6.6) | 4 (2.5) | 4 (4.2) | 12 (30.8) |  |
| Severe | 1 (0.3) | 0 | 1 (1.1) | 0 |  |
| Pulmonary regurgitation |  |  |  |  | 0.726 |
| None | 219 (71.8) | 112 (70.9) | 71 (74.7) | 36 (69.2) |  |
| Mild | 86 (28.2) | 46 (29.1) | 24 (25.3) | 16 (30.8) |  |
| Moderate-Severe | 0 | 0 | 0 | 0 |  |
| Left ventricular systolic impairment grade |  |  |  |  | 0.069 |
| Mild (45-54%) | 14 (4.6) | 12 (7.6) | 1 (1.1) | 1 (1.9) |  |
| Moderate (36 – 44%) | 5 (1.6) | 1 (0.6) | 1 (1.1) | 3 (5.8) |  |
| Severe (≤35%) | 13 (4.3) | 7 (4.4) | 3 (3.2) | 3 (5.8) |  |
| **Management** |  |  |  |  |  |
| Choice of 2^nd^ vasopressor / inotrope |  |  |  |  | 0.833 |
| Vasopressin | 10 (3.3) | 0 | 5 (5.3) | 5 (9.6) |  |
| Adrenaline | 10 (3.3) | 3 (1.9) | 4 (4.2) | 3 (5.8) |  |
| Milrinone | 1 (0.3) | 0 | 0 | 1 |  |
| Dobutamine | 3 (1.0) | 0 | 0 | 3 (5.8) |  |
| Inhaled nitric oxide n (%) | 4 (1.3) | 0 | 1 (1.1) | 3 (5.8) |  |
| Mechanical ventilation duration; days | 16 (10 – 24) | 16 (9 – 24) | 18 (11 – 25) | 12 (6 – 21) | 0.232 |
| ICU length of stay; days | 20 (12 – 30) | 21 (12 – 30) | 23 (12 – 32) | 16 (10 – 26) | 0.051 |
