# Supplementary material for: Cardiovascular subphenotypes in patients with COVID‐19 pneumonitis whose lungs are mechanically ventilated: a single‐centre retrospective observational study
Source: Anaesthesia. 2022 Mar 3;77(7):763–71. doi: 10.1111/anae.15700 (PMC9314994; doi:10.1111/anae.15700)
Supplement: Supplementary file 5 — Table S2. Clinical parameters in eligible patients who did not receive transthoracic echocardiography [file ANAE-77-763-s001.docx]

Table S2: Clinical parameters in eligible patients that did not receive transthoracic echocardiography. Values are number (proportion) or median (IQR).

| Admission parameters | Eligible patients that did not receive transthoracic echocardiography (N=203) |
| --- | --- |
| Age (years) | 57 (45 – 68) |
| Sex n %Male | 140 (69.0) |
| Acute Respiratory Distress Syndrome severity |  |
| Mild | 35 (17.2) |
| Moderate | 156 (76.9) |
| Severe | 12 (5.9) |
| **Clinical** |  |
| PaO2:FiO2 ratio | 23 (19 – 29) |
| C-reactive protein (mg mL^-1^, n=314) | 108 (62 – 192) |
| High sensitivity troponin-I (ng L^-1^, n=206) | 12 (<5 – 20) |
| D-dimer (ng mL^-1^, n=210) | 924 (504 – 1909) |
| Mean tidal volume (mLs kg^-1^ predicted body weight) | 7 (6 – 8) |
| Chest radiography opacification score (0-16) | 8 (6 – 8) |
| Deadspace fraction | 0.7 (0.6 – 0.8) |
| Dynamic compliance (mLs cm H_2_O^-1^) | 31 (20 – 43) |
| Peak inspiratory airway pressure (cm H_2_O) | 25 (18 – 29) |
| Positive end expiratory pressure (cm H_2_O) | 8 (5 – 10) |
| Urine output (mLs kg^-1^ hour^-1^) | 0.7 (0.4 – 1.0) |
| Vasopressor dose | 0 (0 – 0.03) |
| **Management** |  |
| Prone ventilation n (%) | 119 (58.6) |
| Neuromuscular blockade n (%) | 170 (83.7) |
| Renal replacement therapy n (%) | 57 (28.1) |
| 90-day mortality n (%) | 49 (24.1) |
